# Risser patient satisfaction scale: a validation study in Greek cancer patients

**DOI:** 10.1186/1472-6955-11-27

**Published:** 2012-11-29

**Authors:** Andreas Charalambous, Theodoula Adamakidou

**Affiliations:** 1 Nursing Department, School of Health Sciences, Cyprus University of Technology, 15th Vragadinou Streer, Limassol, 3041, Cyprus; 2 Head of the Euro-Mediterranean Research Center for Oncology and Palliative Care, Cyprus University of Technology, Limassol, Cyprus

**Keywords:** Nursing care, Patient satisfaction, Validation, Risser patient satisfaction scale, Cancer settings, Cancer patients

## Abstract

**Background:**

The current healthcare climate is characterized by a constant battle for the provision of quality care with limited resources and with patient satisfaction receiving increased attention, there is a need for reliable and valid assessment measures. This study describes the adaptation, testing and validation of the Risser Patient satisfaction Scale in an oncology care setting in Greece. The rationale for this study lies in the scarcity of such measures in the Greek language.

**Methods:**

This is a test retest validation study in Greece. Data were collected from 298 hospitalized cancer patients. The validation methodology included the assessment of the item internal consistency, using the Cronbach alpha coefficient. The test-retest reliability was tested by the Kappa correlation coefficient**.**

**Results:**

The scale demonstrated very good psychometric properties. The internal consistency of the instrument was good, Cronbach’s alpha was found to be 0.78 (p<0.001) and Kappa coefficient for reproducibility was found to be K=0.89 (95% CI: 0.83-0.91 p<0.0001).

**Conclusion:**

The findings demonstrated strong agreement of the scale, suggesting that the Greek version offers substantial reliability. This study provides a valid and reliable tool to assess patient satisfaction in oncology settings. Means to monitor patient satisfaction, a key aspect of the policy agenda for quality care remain important for nurse leaders to develop better care in oncology settings.

## Introduction

In the health care sector, patient satisfaction has emerged as an important component of the quality of care, and has been used as a means to attain, maintain and monitor it. Despite its popularity and wide acceptability, through time it sparked debates among users and providers of health care services. Mainly these were concentrated on the conceptualization of the term. Therefore, quality of care has often been defined differently among stakeholders, such as employers, insurance companies, health care managers, physicians and patients. Furthermore, the complexity of patient satisfaction has been intensified by the fact that it was related with aspects such as the patient expectations [1,2], health status [3,4], personal characteristics [1,5] and the health care system characteristics [4,6].

Similarly, satisfaction with the hospital experience is a complex and multifactor phenomenon which incorporates (but it is not limited to) relationships with medical personnel, physical surroundings and/or the healthcare organization itself [4,5], requiring a distinction between patient satisfaction with nursing care and other domains of satisfaction [6]. This aspect becomes important when researchers face the dilemma of which questionnaire is more appropriate for measuring an explicit aspect of patient’s satisfaction.

Patient satisfaction is considered a focal concern of quality assurance and it can serve as an outcome measure of the quality of health care and provides a consumer perspective that can contribute to a complete, balanced evaluation of the structure process and outcome of services [2]. Therefore, in order to effectively assess patient satisfaction across different cultural backgrounds it is essential to bear in mind that specific aspects may be taken into consideration differently based on what these cultural norms impose on the patient. Hence, any selected satisfaction questionnaire needs to be previously translated, culturally adapted and validated in the targeted population [7].

## Background

Merkouris et al. [1] comment that patient characteristics, attitudes and prior experiences formed a set of expectations about care which is the standard used by patients for judging the care they receive. Risser [8] associated expectations with perceptions, conceptualizing patient satisfaction as the degree of congruency between what the patient expects and what is offered by the nursing care.

Therefore patient satisfaction can be conceptualized as the patients’ subjective perception of what the caregivers (i.e. nurses) must regard as reality, even though this perception may disregard the appropriateness of therapy and outcomes of the patients’ health status. In addition, patients’ opinions are important because they are the best source of information to the providers in terms of what is important (i.e. for the nursing care), and this is the reason why this information can be used in health care planning and evaluation [4,9].

Schmidt [10] found that a relationship existed between a patient’s perception of nursing care and the patient’s overall level of satisfaction during the hospital experience. The nurse is at the forefront of the care provided at the hospital, is responsible to provide direct care to patients, to organize and coordinate the care with other hospital services and comprises the major part of the health care staff [3]. However, patient’s perception of satisfaction or dissatisfaction is not always merely a reflection of the nursing care provided [11]. Patients have difficulties in dissociating their satisfaction with nursing care from their overall hospital experience satisfaction [12]. So, it is of crucial importance that all health care professionals co-operate to improve care quality, in collaboration with the care-receivers [4,13].

Preceding studies demonstrated high patient satisfaction with nursing care [14] which is related to good administrative support for nursing care, good relationships between nurses and physicians, and adequate staff numbers [15]. Dissatisfaction or simply lack of satisfaction was associated to the lack of nursing control services [14,16], nurse burnout [15], decrease of nursing staff [14,17] and the inadequate amount of information provided by nurses [18].

Nowadays, nursing care is recognized as an area of health care where the patient is seen both as a client and as a consumer of health care services [4]. Nursing evidence-based research and knowledge is needed to support the vital role they play in providing quality care to patients [19]. So, it is crucial for nursing to develop valid and reliable instruments to measure patient satisfaction [13,20].

Abdellah and Levile [21] back in the 50s developed the first instrument to measure patient satisfaction. More than 2 decades later, Risser [8] developed one of the first instruments, the Patient Satisfaction Scale–PSS, to measure patient satisfaction explicitly to the nursing care in the outpatient setting, incorporating three distinct dimensions of the care.

Although, there are several tools in the Greek language to estimate patient satisfaction with the overall care or the nursing care explicitly [14,22,23], to the best of our knowledge there are no tools that address the care provided to cancer patients explicitly. Therefore, the decision to validate the PSS questionnaire was merely drawn on the fact that there is always room for new scales in Greek which can capture an aspect that previously was left unexplored or understudied. This perspective on the necessity of satisfaction scales in the enhancement of patients’ outcomes evaluation has been stressed by Apolone and Mosconi [24] and this study comes as a response to the need to adapt, test and validate questionnaires for patient satisfaction in Greek.

This paper describes the translation and psychometric validation of the PSS in hospitalized Greek cancer patients. The PSS has satisfied all of Rubin’s criteria [25], for comprehensive content; multi-item subscales; a uniform response scale; at least four response options for each item; interpretability using norms or other criteria and its validity assessment is important to accurately measure quality of care; it is a very popular tool for eliciting satisfaction in different clinical settings and has previously been validated to the Cypriot population showing that it is a practical tool to measure patient satisfaction in oncology settings [26]. This study will allow cross-cultural adaptation and validation of the Greek-language version of the questionnaire in the Greek population with its distinctive cultural influences.

This study was guided by the following research questions:

a) What are the psychometric properties of the Greek Version of the Risser Patient Satisfaction Scale?

b) Does the Greek Version of the PSS offers substantial reliability?

## Methods

### Setting and sample

The study was conducted in a large Anticancer Hospital in Athens. Potential eligible participants were identified prior to running the random number selection program based on a set of pre-determined inclusion and exclusion criteria. The inclusion criteria included adult cancer patients 18 years or older who were receiving care at the hospital for at least 48 h. The potential participants needed to be able to speak and understand Greek and they had accepted to provide a written informed consent. No restrictions were imposed in relation to the type of cancer. Finally prospective eligible patients should have a score of >50 on the Karnofsky Performance Scale Index [27] and a mean of >50 on the Attentional Function Index (AFI) which was used to measure perceived cognitive function [28]. Patient's performance was assessed by a research assistant and the AFI was assessed by the patients themselves prior to running the random number selection program. The results of these assessments varied for both scales. For the Karnofsky Scale the results ranged between 60 and 80 with the level of 60 indicating that the patient “Requires occasional assistance, but is able to care for most personal needs” and the level of 80 indicating that the patients has “Normal activity with effort; some signs or symptoms of disease” [27]. The AFI produced scores ranging from 55–82. Patients who score 50 to 75 function moderately well and patients who score >75 function well [29].

The exclusion criteria included patients at a terminal phase of the illness (receiving palliative care), impaired cognitive ability, patients diagnosed within the last 6 months and quarantined patients (transplantation, infections).

A random number selection program was used for the selection of the patients over a period of 3 months (extending from November 2010 to January 2011). The sampling frame that was employed included consecutive patients admitted in the whole hospital. Out of the 326 patients that were identified as potential participants during the selection process, 28 patients were further excluded for various reasons: 3 (0.92%) refused to participate in the study, 5 (1.55%) had brain metastasis with impaired cognitive ability (deteriorated after admission), 10 (3.06%) received an early discharge or transfer to another hospital (cared for less than 2 days in the hospital), 4 (1.22%) had communication problems and 6 (1.84%) were in the terminal stage of the illness. Therefore, the final sample consisted of 298 participants who all agree to answer the questionnaire (response rate 100%).

Patients were invited to participate in the research, after receiving detailed oral and written explanations in relation to the study’s objectives by the researchers in face to face meetings and signing an informed consent. Potential participants were encouraged to address any questions or/and concerns to the researchers with regards to their participation.

Each patient completed the self-administered Greek version of the PSS and repeated his/her answers after a four week period (re-test). Upon discharge, the participants were provided with stamped envelopes and the anonymous questionnaire (with a pairing code), and were asked to post it after 4 weeks. The participants provided oral consent to the researchers with respect to phone or text (sms) reminders. Therefore, weekly call and/or text reminders were made to the participants that did not send their responses in order to assure a high response rate. This was the means by which the reliability of the questionnaire was assessed. Of the 298 patients provided with a re-test survey, 253 (85%) completed this second questionnaire.

### Questionnaire description

A modified version of the Risser [8] Patient Satisfaction Scale (PSS) was used to elicit the research data. The version implemented here is the one produced by Hinshaw and Atwood [30] which compared to the original Risser scale differs at the 7^th^ item of the “technical-professional” subscale where the phrase “over the telephone” was deleted. This version was psychometrically tested in five studies with a total of 600 patients, primarily medical-surgical inpatients. The results showed stable internal consistency estimates in the different studies with the average coefficients alpha values reported being 0.79, 0.78 and 0.88 for the three subscales respectively. The PSS was designed to evaluate patients’ attitudes towards nurses and nursing, and originally contained three subscales with a total number of 25 items (Table 1) defined as follows:

1. Technical-Professional (TP) domain contains seven items concerning technical issues on care and measurement of the nurses’ behaviors;.

2. Educational Relationship (ER) domain contains seven items concerning nurses’ attitude with patients, the exchange of information between the nurse and patient; and

3. Trusting Relationship (TR) domain approaches eleven interpersonal relationship situations between nurses and patients the verbal and nonverbal communication that occurs between the nurse and client [8,26].

**Table 1:** Patient satisfaction mean by subscale and by item

**Items by subscale**	**Mean**	**SD**
**Technical- professional**	**3.11**	
The nurse is skillful in assisting the doctor in various procedures	3.22	(1.44)
The nurses really knows what she is talking about	3.83	(1.57)
The nurse is not precise in doing her work	1.28	(0.56)
The nurse makes it a point to show me how to follow medical instructions	4.06	(1.42)
The nurse is too slow to do things for me	1.69	(1.02)
The nurse is often too disorganized to look on top of things	2.70	(1.21)
The nurse gives good advice	4.49	(1.36)
**Interpersonal-educational**	**2.90**	
The nurse gives directions at the right speed	2.29	(1.60)
The nurse asks a lot of questions but once she finds the answers, she doesn’t seem to do anything	3.61	(1.40)
I wish the nurse would tell me about the results of my tests more than she does	3.15	(1.62)
The nurse explains things in simple language	3.80	(1.57)
It is always easy to understand what the nurse is talking about	1.40	(1.02)
Too often the nurse thinks you can’t understand the medical explanation of your illness, so she just doesn’t bother to explain	1.13	(1.97)
The nurse always gives complete enough explanations of why tests are ordered	3.89	(1.76)
**Interpersonal-trusting**	**4.05**	
The nurse is understanding in listening to a patient’s problems	3.70	(1.90)
The nurse should be more attentive than she is	3.95	(1.49)
The nurse is just not patience enough	4.10	(1.23)
When I need to talk to someone, I can go to the nurse with my problems	3.54	(2.06)
The nurse is too busy at the desk to spend time talking with me	4.10	(1.14)
The nurse is pleasant to be around	4.55	(1.54)
I am tired of the nurse talking down to me	4.35	(0.97)
The nurse is a person who can understand how I feel	2.98	(1.74)
A person feels free to ask the nurse questions	4.25	(0.70)
The nurse should be more friendly than she is	4.63	(0.95)
Just talking to the nurse makes me feel better	4.09	(2.23)
**Total Scale**	**3.20**	

Both positive and negative sentences were included in each subscale. Each question is assessed on a five point Likert-type measurement scale ranging from “Strongly agree” (=1) to “Strongly disagree” (=5). The negative sentences are assessed in reverse, and the higher the PSS score is, the higher is the patient satisfaction with the nursing care provided [26].

### Translation and cultural adaptation

Part of this study was the translation and cultural adaptation of the Risser questionnaire in the Greek language. Although, a Greek version was available, the translation and adaptation occurred in a different population (Greek-Cypriot). Although “Greek-Greek” vernacular differs from “Cypriot-Greek” vernacular several changes were necessary in order to adjust the questionnaire to the Greek (Athenian) patients. For example the question “The nurse is understanding in listening to a patient’s problems”, this was translated differently in the two populations in order to achieve the same meaning. Explicitly in the Greek-Cypriot version the question was translated as «*Η νοσηλεύτρια επιδεικνύει κατανόηση στα προβλήματα του ασθενή*» and in the Greek-Greek version was translated as «*Η νοσηλεύτρια κατανοεί τα προβλήματα που αντιμετωπίζει ο ασθενής*».

In order to produce an adapted questionnaire of the highest semantic equivalence it is important to follow internationally recommended criteria suggested by the relevant literature [31,32]. Therefore, the adaptation process was based on the Minimal Translation Criteria [33] that included translation and back translation of the original questionnaire. Three independent bilingual nurses with previous experience in translating questionnaires produced the English to Greek translation. Subsequently, the questionnaire was back-translated in Greek by three independent bilingual nurses. The produced English versions of the questionnaire were compared with the original one, and this process identified some problematic questions. These were addressed by revising the questions based on the translators’ mutually agreed suggestions. Following the translation and back-translation, a cognitive debriefing process was used to identify any problems with language and to assess the degree to which a respondent’s understanding of each item matched the content that it was meant to elicit. The Cognitive Debriefing in this study, formed a part of the translation process, and included cognitive debriefing interviews with 9 bilingual professional oncology nurses with experience in the translation of instruments and 1 professional translator. These experts were invited to review the translated version of the PSS. They reviewed the questionnaire and were asked specific questions by the researchers as to whether the translation was both culturally and linguistically correct. Furthermore, they were asked to acknowledge whether the wording in the questionnaire was clear and unambiguous. There were no suggestions made to adjust or change the wording of the translated Greek version by the Cognitive Debriefing process. The translated version of the scale was then administered to a convenienne sample of 15 patients during their hospitalization at an oncology setting. The participants answered the scale’s questions by themselves without any difficulties in understanding the meaning. They found the scale concise, easy to understand and easy to complete. Finally, the pretest and reliability testing (test–retest) followed.

#### Ethics

All ethical guidelines recommended by national and international ethics committees were applied in the study. The study had a voluntary nature of participation and the participants’ confidentiality and anonymity were maintained throughout the study. The study’s protocol was reviewed and approved by the St. Savvas Oncology Hospital Ethics Committee (Athens-Greece).

#### Data analyses

The Internal consistency and reproducibility were measured as part of the reliability testing of the translated tool. The same psychometric tests as in the Charalambous [26] study were applied in order to examine the internal consistency of the three subscales namely: Cronbach’s coefficient alpha, inter-item, item-subscale and subscale-subscale correlations.

According to the international literature a desired or adequate level for coefficient alpha is 0.70 or above [34] even if this criterion level according to [35] should be considered in the light of its dimensionality or construct validity.

The homogeneity ratio (Scott’s Homogeneity Ratio) [36] represents the degree to which the actual total score variance exceeds the variance that would be obtained with uncorrelated items, in ration to the maximum difference that would be found if all items were perfectly correlated [37,38].

The Kappa coefficient (k) was applied for evaluating the test–retest reliability [39]. This coefficient has values ranging from −1 to + 1. A value of 1 implies perfect agreement and values less than 1 imply less than perfect agreement [40,41]. There are several standards for strength of agreement for the kappa coefficient in the literature [41-43]. However perhaps the most prominent is the ones introduced by Landis and Koch [44]: ≤0=poor, .01–.20=slight, .21–.40=fair, .41–.60=moderate, .61–.80=substantial and .81–1=almost perfect.

With the criterion validity statistical test, the researcher can explore whether an instrument reflects a certain set of abilities or used to demonstrate the accuracy of a measure or procedure by comparing it with another measure or procedure which has been demonstrated to be valid [45,46]. For this study the concurrent validity of the questionnaire was tested. Predictive validity occurs when the criterion measures are obtained at a time after the test scores [47].

In order to study the structural validity of the questionnaire, the researchers applied a Varimax (oblique) rotation and subsequent Cronbach’s alpha was carried out on the 298 questionnaires. The rationale for implementing the rotating factors comes from Thurstone [48] and Cattell [49] who defended its use because this procedure simplifies the factor structure and therefore makes its interpretation easier and more reliable. In order to analyze the data, we used the *SPSS* (Statistical Package for the Social Sciences) version 17.0 software for Windows.

## Results

The sample consisted of 133 (44.6%) men and 165 (55.4%) women. Most of the participants belonged to the age group of 61–70 years old and 198 (66.4%) lived in Athens. Gastrointestinal cancer was the most common diagnosis (23.2%), 158 (53%) of the patients were hospitalised for 2–10 days and for the 208 (69.8%) participants this was their first hospitalization. A more detailed description of the sample’s sociodemographic characteristics appears in Table 2.

**Table 2:** Demographic and clinical characteristics of the patients

		**Ν**	**%**
**Gender**	Men	133	44.6
	Women	165	55.4
**Age Group**	20-30	15	5.0
	31-40	19	6.4
	41-50	44	14.8
	51-60	66	22.1
	61-70	78	26.2
	71-80	65	21.8
	>80	11	3.7
**Place of Residence**	Athens	198	66.4
	County	100	33.6
**Diagnosis (Cancer site)**	Breast	54	18.1
	Respiratory system	26	8.7
	Urinary system	49	16.4
	Gastrointestinal system	69	23.2
	Melanoma	17	5.7
	Genital system	40	13.4
	Other	43	14.4
**Days of hospitalization**	2-10	158	53.0
	11-20	114	38.3
	21-30	11	3.7
	31-40	7	2.3
	>40	8	2.7
**Previous hospitalization**	No	208	69.8
	Yes	90	30.2

The coefficient alpha was found within the internationally recommended criterion levels (α>0.70) for all three subscales. The Interpersonal-trusting subscale demonstrated the highest alpha coefficient. The inter-item was r=0.40 and the item-subscale was r=0.58 that were consistent with earlier studies [8,50].

The coefficient alpha for the Interpersonal-Educational subscale was estimated at α=0.79 which was the second highest. The Technical-Professional subscale showed a coefficient alpha α=0.77. The inter-item and inter-subscale correlations for the Interpersonal-Educational and the Technical-Professional sub-scale confirmed the alphas.

One way of assessing the consistency of the total instrument is through the exploration of the intercorrelations among the consisting subscales. In this study we carried out a subscale to subscale correlation matrix (Table 3). The findings point out that the correlations found (r=0.57-0.69) are consistent with internationally established acceptable criteria (r=0.55–0.70) [51]. These indicate a medium to high correlation coefficients between the subscales. In order to examine the possible combination of all items as one scale with three sub-divisions the item-subscale correlations were calculated. Drawing on the findings of preceding studies [8,30,50] and the recommendations by Charalambous [26] inter-item correlations needed to average r = 0.30 to 0.70 to be high enough to index similar content. Therefore, the calculations of medium to high (r=0.41-0.65) inter-item correlations found here suggest the combination of all items as one scale with three subsets of content areas of same attitude.

**Table 3:** Subscale to subscale correlation (Pearson correlation)

**Subscales**	**Technical-professional**	**Interpersonal-educational**
Interpersonal-educational	0.64	
Interpersonal-trusting	0.57	0.69

The Scott’s Homogeneity Ratio calculations appear in Table 4. A coefficient between 0 and 1 should be produced by this statistical test. The optimal level is above 0 but less than 1, since 1 would indicate an inefficient index where only one item would represent the attributes as well as the set of items [52]. The findings showed that all items demonstrated Scott’s Homogeneity Ratio above 0 but less than 1. Therefore, in relation to Scott’s analysis the scale conforms to the assumption that most scales are likely to have homogeneity ratios of 0.2 to 0.3 [53].

**Table 4:** Internal consistency reliability coefficients and homogeneity ratios

**Satisfaction subscales**	**Questions**	**Cronbach *α***	**Scott’s homogeneity ratio**
Technical-Professional	1-7	0.77	0.35
Interpersonal-educational	8-14	0.79	0.37
Interpersonal-trusting	15-25	0.80	0.49
Total *α* *		0.78	0.35

One of the issues that needed to be clarified was the structure of the translated version of the PSS and whether this structure was equivalent to the one produced in the original study. In order to examine this, a factor analysis was performed using the Bartlett’s test of sphericity [54] and a Kaiser-Meyer-Olkin (KMO) Measure of Sampling Adequacy [55]. The significance levels were set to p<0.05 for the Bartlett’s test of sphericity and >0.6 for the Kaiser-Meyer-Olkin (KMO) Measure of Sampling Adequacy. The factors were considered as important if its eigenvalue exceeded 1.0.

The statistical analysis produced an overall Cohen’s kappa coefficient (K=0.89) for reproducibility (95% CI: 0.83-0.91 p<0.0001) of the scale. According to the Landis and Koch [44] classification this is considered as “almost perfect”. On the matrix produced, the majority of the items (96 items) demonstrated very good reproducibility (K>0.88), with only 9 items having moderate reproducibility (K=0.45-0.58). Two of the items were found to have fair or low reproducibility (K<0.41). The reproducibility findings for this study by subscales were also very good, as illustrated in Table 5.

**Table 5:** The re-test reliability by subscale

**Subscale**	**Coefficient alpha**	**95% Confidence interval**	**P**
Technical-professional	0.77	0.79-0.91	<0.001
Interpersonal-educational	0.79	0.85-0.94	<0.001
Interpersonal-trusting	0.80	0.88-0.96	<0.001

The data analysis demonstrated that the translated version of the scale has significant criterion validity with regards to the three domains of the scale. This conclusion lays on the statistically significant correlations found between respondents’ trusting (r=0.20-0.35, p –values 0.025-0.039), educational (r=0.32-0.35, p –values 0.015 - 0.031) and professional (r=0.35-0.49, p –values 0.010 - 0.025) relationships on each of the research items and the respondents’ previous admissions.

Overall, the individual scores in the study were positively skewed. The positively skewed attitude toward nursing care found here is consistent with other studies of attitudes toward nurses [5,56,57].

## Discussion

This study has provided further validation of the Risser Patient Satisfaction Scale, a popular scale that received extensive attention world-wide. The psychometric testing of the Greek version of the Risser Patient Satisfaction Scale came as a response to the increased need of integrating valid satisfaction scales in daily practice in Greek oncology settings. The current study presents a cultural adaptation of the Greek version of the PSS, following internationally accepted methodological procedures. These procedures were consistent with the methodology used for adapting the scale in other languages [58-60].

As patients had a number of different nurses caring for them, they had problems answering questions that referred to all nurses. In addition, cancer nursing occurs within a multidisciplinary or interdisciplinary context and patients had difficulties to isolate the nursing care from the whole health care experience. Therefore, this aspect raises concerns if indeed patients perceived being satisfied or dissatisfied based on the nursing interventions and interactions. The lack of sensitivity and the difficulty of the patients to distinguish nursing care from their overall experience with health care posses as a threat to the validity of these measurements [61,62]. However, this is not a new problem but rather an aspect that satisfaction scales have failed to address over time [63].

Despite these longstanding sensitivity issues of patient satisfaction scales, the study’s findings revealed that the Greek version of the Risser Patient Satisfaction Scale is a valid, comprehensive and reliable tool that is appropriate to elicit data on cancer patients’ satisfaction with the received nursing care. The total Alpha coefficient as well as the individual alphas of the subscales are >70 that signify a very good reliability of the scale. The reported alphas are well above the international recommended minimum criterion and comparable to the previous validation studies [26,58-60]. The scale also demonstrated significant criterion validity that coincides with those found in the Cypriot study [26].

As the economic and social climates continue to favor competition in healthcare, patient satisfaction will remain an important factor for attracting and maintaining patients. It is clear that the process of evaluation and thus the meaning of patient satisfaction data are highly dependent upon the role in which patients perceive themselves in relation to the health care system.

The PSS Scale has been used world-wide across different cultures. It has been used in different non-English speaking countries and translated into several languages. The scale has been previously translated in Greek, however the adaptation and validation was tested in Cypriot population and not Greek. Despite the many apparent commonalities between the population of the two countries there are also not so apparent discrepancies that call for suspicion when validated instruments in one country are about to be used in the other and vice versa. Therefore it was considered necessary by the researchers to adapt this scale explicitly for the Greek population and the results verified their decision as differences were found between the two available Greek versions.

### Limitations of the study

The Greek version of PSS demonstrated psychometric properties comparable to those reported for the original version in other European countries; however, a number of limitations need to be acknowledged for this study. The research study was undertaken in single anticancer hospital in Athens. However, taking into consideration that similar conditions exist in the other three anticancer hospitals, it is possible that the findings can be generalizable. Moreover, the fact that this was a validation study and not a study aiming to actually measure patients’ satisfaction, generalizability is not really an issue nor it posses a threat to the findings. Another limitation was that patients may have felt constrained in their responses if they perceived that this information may be provided to their healthcare providers [64]. For the same reason, patients might have favoured the positive responses to the questions. According to the study protocol, the questionnaire was distributed only to patients based on pre-determined inclusion and exclusion criteria, therefore, patients with a different background might have responded differently to the questionnaire.

## Conclusion

The psychometric properties and the linguistic equivalence of the translated version of the PSS demonstrate that this scale is not only an acceptable and reliable measure of patients’ satisfaction within the context of Greece but it is also compatible with the original version as well as the other translated versions of the scale. This allows for possible cross-sectional and cross-cultural comparisons in relation to the patients’ satisfaction among different countries.

## Competing interests

The authors declare that they have no competing interests.

## Authors’ contributions

The conception of this study lies with AC as well as the design, analysis and interpretation of data. TA has participated in the data collection process. All authors have been involved in drafting the manuscript and have given final approval of the version to be published.

## Pre-publication history

The pre-publication history for this paper can be accessed here:

http://www.biomedcentral.com/1472-6955/11/27/prepub
